# Cu Nanoparticle-Based Solution and Paper Strips for Colorimetric and Visual Detection of Heavy Metal Ions

**DOI:** 10.1021/acsomega.2c03687

**Published:** 2022-10-09

**Authors:** Trilochan Baral, Chitraniva Datta, Subhojit Das

**Affiliations:** † Department of Chemistry, National Institute of Technology Agartala, Tripura799046, India

## Abstract

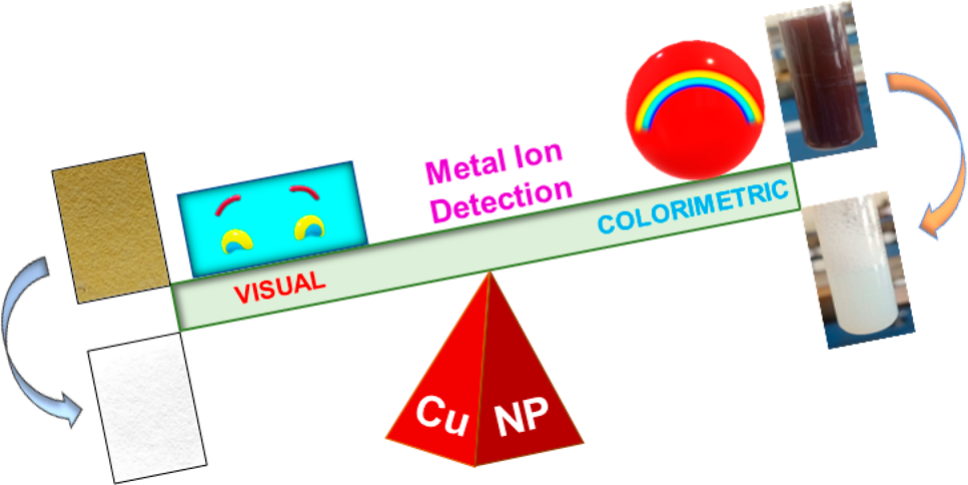

The intrinsic toxicity of heavy metal ions to human health or other species calls for the need to develop an analytical tool for the easy and rapid detection of these ions based on inexpensive and stable nanomaterials. This article describes the potential utility of stable Cu nanoparticles (CuNPs) in the detection of toxic metal ions by solution and paper strip-based methods. For this, first, a dodecyl sulfate ion-stabilized CuNP (DS-CuNP) colloid was synthesized by a chemical reduction method. This was followed by treating the dispersion with heavy metal ions and monitoring the spectral change by spectrophotometric and colorimetric techniques. Among a host of metal ions, Hg^2+^, Cd^2+^, and Pb^2+^ have been found to significantly affect the surface plasmon resonance band of CuNPs by concomitantly altering the color of its solution. Notably, the brownish color of CuNP solution changed readily to milky white in the presence of Hg^2+^. Furthermore, the fabricated brownish-yellow test paper strips containing DS-CuNPs transformed to a prominent white color in the presence of a few drops of Hg^2+^ solution. This change in color of the paper strips could be visually detected by the naked eye. The experiments involving the detection of the various ions were carried out by optimizing the experimental conditions qualitatively as well as quantitatively. The limit of detection of the analytes (metal ions) has been found to be 10 μM. Routine analytical techniques like UV–vis spectroscopy, dynamic light scattering, transmission electron microscopy, and Fourier transform infrared spectroscopy formed part of the experiments.

## Introduction

Heavy metals, particularly lead, cadmium, and mercury, pose serious risks to both the human body and the environment.^1−5^ These metals have, therefore, been thoroughly investigated, and international organizations such as the World Health Organization (WHO) examine their effects on human health on a regular basis. For instance, the general populace is primarily exposed to mercury via food, of which, fish is a major source of methylmercury exposure. It is believed that methyl mercury accumulates in the human body and destroys a variety of organs.^3^ Further, claims have also been made that mercury from dental amalgams cause a variety of diseases. Again, poor recycling of Cd-containing goods, such as Ni–Cd rechargeable batteries, which are being thrown away with domestic wastes, forms the primary source of Cd exposure.^4^ Lastly, human beings are exposed to lead through lead emissions from gasoline. It is anticipated that lead has neurotoxic effects at lower levels of exposure than previously thought.^5^ Based on the above facts, it may be stated that the detection of the mentioned ions becomes pertinent while also simultaneously rendering them harmless.

Until now, many methods for detecting heavy metal ions have been reported. Among them, the colorimetric assay of metal ions is increasingly popular. This is primarily because colorimetric approaches are convenient in most applications, as they can be easily determined by color change that can be visualized with the naked eye without requiring the use of any special equipment.^6−12^

Environmental nanotechnology, which is arguably the most recent application of nanomaterials, is currently being used as novel instruments in environmental sensing and biomonitoring, pathogenic bacteria capture, wastewater treatment, and other applications.^13^ Colloidal nanoparticles (NPs) feature unique structural and optical properties, including quantum size effects, surface plasmon resonance (SPR), and high surface-to-volume ratio, making them suitable platforms for a wide range of materials applications.^14,15^ The detection of analytes utilizing nanomaterials is often based on a molecular interaction between the specified analytes and the surface of the NP, which is functionalized with appropriate surfactants. As label-free systems, NPs have excellent chemical and biological sensing capabilities. The availability of the finest colloidal metal nanostructures with finely modified surfaces makes them amenable for detection with high selectivity and sensitivity.^15^ Among the metal NPs, CuNPs are interesting candidates for biomedical applications,^16^ particularly biosensing, due to their visible SPR spectra and fluorescence features with a favorable quantum yield.^17−21^ Because CuNPs are prone to surface oxidation, engineering the surface passivation by tweaking the synthesis methods becomes appealing to keep them from oxidation.^22−25^ This makes surface-modified CuNPs promising candidates for colorimetric and fluorescence-based detection of analytes.^19−21^

**Scheme 1:**
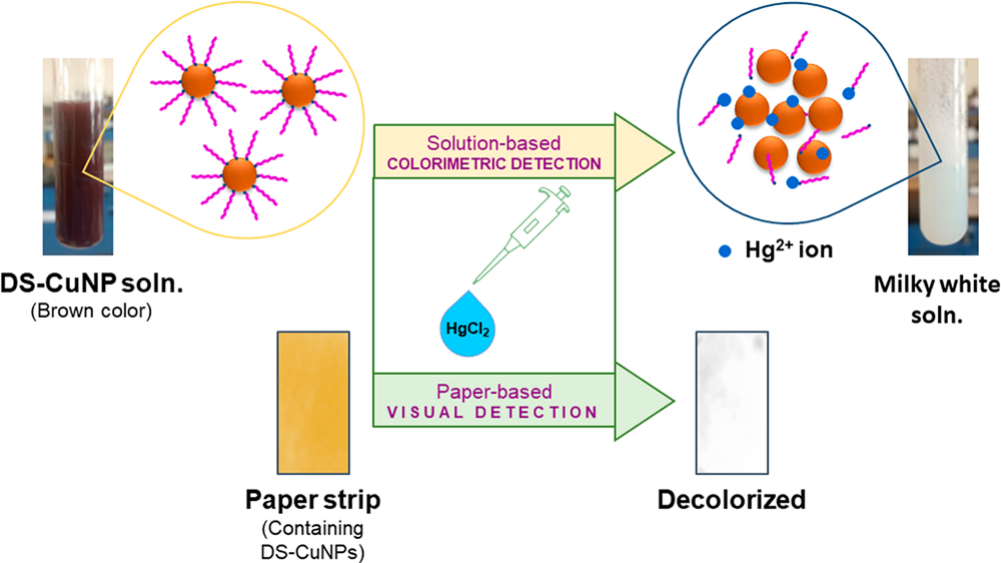
Illustration of the Detection of Hg^2+^ (a Heavy Metal Ion) Using Dodecyl Sulfate Ion-Stabilized CuNPs (DS-CuNPs) Contained in Solution and Paper Strips

Therefore, it becomes relevant to design a stable NP system having the potential to detect heavy metal ions at their minimum concentration (conc) levels and alter them to harmless substances. The designed platform should essentially be stable, cost-effective, and readily available. In this article, we report the synthesis of dodecyl sulfate ion-functionalized CuNPs—the resulting colloid being referred to as DS-CuNPs—and its use in the detection of heavy metal ions (as shown in Scheme 1). Dodecyl sulfate ion, an anionic part of the surfactant sodium dodecyl sulfate (SDS), could prevent the CuNPs from oxidation and aggregation by interacting with the NPs through its hydrophilic end. The sensing system made up of DS-CuNPs could conveniently be used for the detection of heavy metals such as Hg^2+^, Cd^2+^, and Pb^2+^ being made possible by monitoring a change in the SPR band of DS-CuNPs in the presence of variable concentration of the analytes. Furthermore, we fabricated paper-based strips containing DS-CuNPs for easy and rapid visual detection of ions over a range of concentrations. This has been demonstrated using Hg^2+^ ions.

## Experimental Section

### Materials and Chemicals

All the chemicals used for the experiments were of reagent grade, procured from Merck, Aldrich, and SD Fine Chemicals. Copper chloride (CuCl_2_·2H_2_O; molecular weight (MW) 170.48 g/mol), sodium dodecyl sulfate (C_12_H_25_NaO_4_S; MW 288.38 g/mol), hydrazine hydrate (N_2_H_5_OH), sodium hydroxide (NaOH), and all other metal salts were used as received. Distilled water was used in all experiments. The details of characterization of various materials have been provided in the Supporting Information.

### Synthesis of SDS-Stabilized CuNPs

CuNPs were synthesized by the chemical reduction method using dodecyl sulfate ion as a surface passivating agent, following an earlier reported protocol.^26^ Approximately 0.085 g of Cu precursor was added to 40 mL of water (conc ≈ 10 mM) in a 250 mL round-bottom flask and allowed to stir under a refluxing condition. This was followed by the addition of 10 mL of SDS solution containing 0.10 g of the surfactant. The solution was then allowed to boil. To the boiling solution, 0.8 mL of 50 mM NaOH solution was added. Then, about 0.4 mL of hydrazine hydrate solution was added in a dropwise manner. The reaction was monitored by rapid changes in color from light blue to deep red colloidal sol. Keeping the conditions unchanged, the reaction was allowed to continue for an additional 20 min in order to ensure that complete reduction of Cu(II) ions to Cu(0) had occurred. The colloid thus obtained was probed for the formation of NPs by regular characterization techniques, and a dilute solution of it was used for further experiments.

### Spectrophotometric/Colorimetric Determination of Metal Ions

For the sensing and quantification of metal ions, to 3 mL of colloidal DS-CuNP solution, definite volumes of metal salt solutions having concentration 1 μM were added after fixed time intervals, and the absorbance of the mixed sample was noted in the range of 500–800 nm. It may be noted that dilute solutions of metal salts were prepared by the serial dilution of stock solution, in order to analyze the sensing limit of CuNPs.

### Fabrication of Test Strips for Visual Detection of Hg^2+^ Ions

For the fabrication of test strips, we used Whatman filter paper. The paper was cut into rectangular-shaped strips (1 cm × 3 cm), and each was immersed into DS-CuNP solution and placed under vacuum overnight. Initially, a brownish-colored paper was obtained, due to CuNP coating, which on drying in ambient air turned to brownish-yellow. The resultant paper strips were used for the detection of Hg^2+^. For this, 1 mL of the Hg^2+^ solution (1 μM) was taken, and the test strip was dipped into it for about a minute. The change in color of the strip was noted for a range of concentrations of Hg^2+^ in the sample. The selectivity of DS-CuNP test strips toward Hg^2+^ ion was assessed by testing other cations such as the likes of Cd^2+^, Pb^2+^, Zn^2+^, and Ca^2+^, among others. The images of the paper strips at different stages were captured using a cell phone camera.

## Results and Discussion

**Figure 1:**
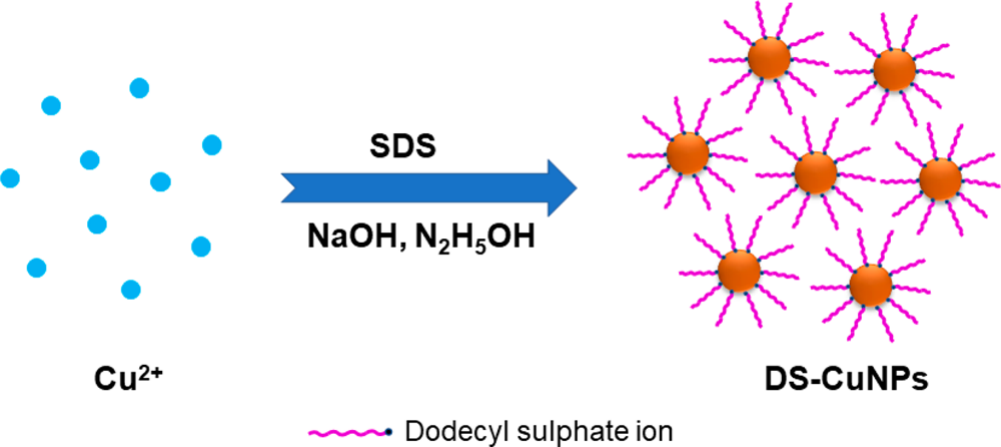
Schematic illustration of the synthesis of DS-CuNPs.

A reddish-brown colored colloid was readily synthesized starting from copper(II) chloride using hydrazine hydrate as a reducing agent and dodecyl sulfate ion as a stabilizing agent. The method used for the synthesis of CuNPs takes a clue from an earlier report.^26^ The synthesis was favorable when the medium was faintly basic. It is believed that stabilization of the CuNP colloid was achieved by the hydrophilic part of the surfactant interacting with the particles, while the hydrophobic tails suffering steric repulsion, thereby keeping the NPs dispersed. A slightly higher concentration of the surfactant was used in the above synthesis to ensure the formation of a stable dispersion. The reddish-brown color indicated the possible formation of DS-CuNPs (Figure 1).

**Figure 2:**
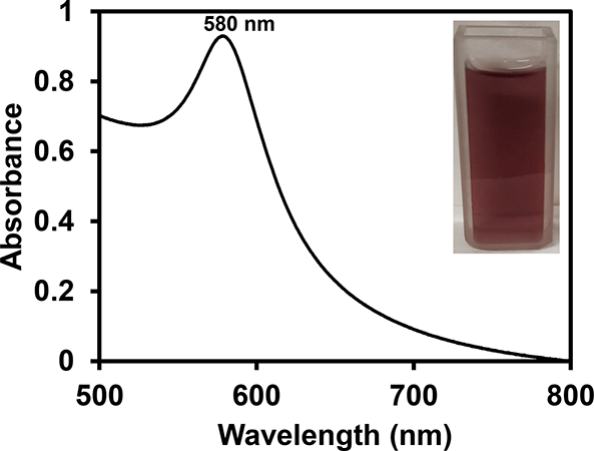
UV–Vis spectrum of DS-CuNP colloid. The image alongside is the corresponding colloidal sol.

As a preliminary investigation tool, UV–vis spectral studies were carried out to establish the formation of a CuNP colloid. The UV–vis spectrum showed the evolution of a band in the region of 580 nm (Figure 2). The appearance of this band is attributed to the SPR of CuNPs.^27^ The color of the colloid and the SPR band position suggested the formation of spherical CuNPs. In addition to the position, the band features a sharp peak, which is indicative of the possible formation of NPs with a narrow size distribution.

**Figure 3:**
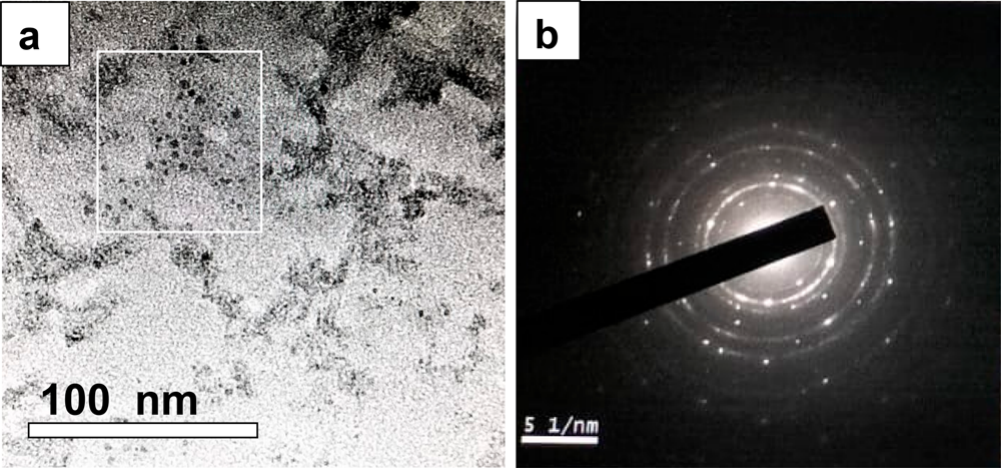
(a) TEM image of DS-CuNPs and (b) SAED pattern of CuNPs.

In order to understand the nature and morphology of the colloidal particles, a transmission electron microscopy (TEM) investigation was carried out. TEM images of colloidal DS-CuNPs can be found in Figure 3a and Figure S1, Supporting Information. It could be observed that the particles are spherical in shape and nearly of the same size. From the TEM images, the average size of NPs determined by taking an ensemble of around 50 particles was about 7 ± 0.4 nm. Further, that the particles were crystalline in nature was investigated from selected area electron diffraction (SAED) pattern, shown in Figure 3b. The SAED pattern was obtained by focusing an electron beam on a region of a collection of particles. Analysis of the diffraction pattern revealed the presence of planes (111), (200), and (220), and these are due to the face-centered cubic (fcc) crystal lattice of CuNPs.

**Figure 4:**
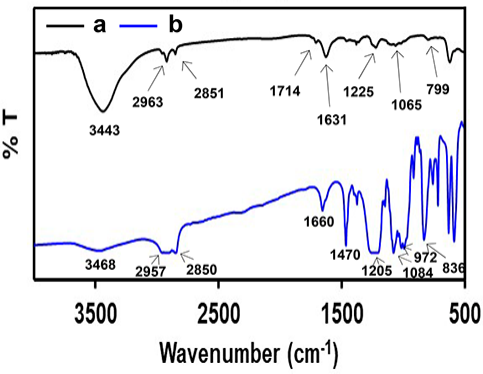
FT-IR spectra of (a) DS-CuNPs and (b) SDS only.

Because CuNPs are susceptible to oxidation at room temperature, effective protection of the NPs in an aqueous solution is crucial before they are used. As such, functionalization of NPs was carried out using the surfactant SDS, wherein the anionic moiety plays a role in binding with NPs. In order to understand the mode of interaction of the surfactant ion and CuNPs, Fourier transform infrared (FTIR) spectroscopic studies were carried out. The specific attachment of the dodecyl sulfate ion on the surface of CuNPs was assessed by comparing the FTIR spectra of DS-CuNPs with that of dodecyl sulfate ion (Figure 4). The spectra of both samples was found to be nearly identical, with apparent shifts in the stretching frequencies of certain specific bonds. Spectral changes were observed for SDS associated with CuNPs with respect to pure SDS. The stretching vibration ν(S=O) showed a shift from 972 to 1065 cm^–1^, while ν(SO_3_) shifted from 1205 to 1225 cm^–1^. The shifts occurred to a higher-frequency side on going from SDS to SDS-CuNPs. Further, for the C–H bond, the asymmetric stretching frequency showed a change of several wavenumbers from 2957 to 2963 cm^–1^, while the symmetric stretching appearing at 2850 cm^–1^ remained barely unchanged in the DS-CuNP sample. This interpretation of observed spectral features of both samples is in alignment with the findings of a previous report.^28^ According to the FTIR investigations, the coordinative attachments between the O-atoms of sulpho groups of SDS and the surface of the NPs allow the anionic moiety to stabilize the resulting CuNPs.

It is worthwhile to consider the mechanism of coordination between SDS and CuNPs. SDS is known to exist in the form of micelles in an aqueous medium, with the long hydrophobic chains pointed toward the core and the hydrophilic heads directed to the outer surface. The sulfate groups on the outer surface of micelles can efficiently coordinate with CuNPs thereby imparting stability to the NPs.^28^ Additionally, the electrostatic repulsion among the ionized SDS micelles plays a role in the formation of small, evenly distributed CuNPs. The interaction between the dodecyl sulfate ion and CuNPs can prevent the particles from increasing their size during the growth as well as from undergoing aggregation.

**Figure 5:**
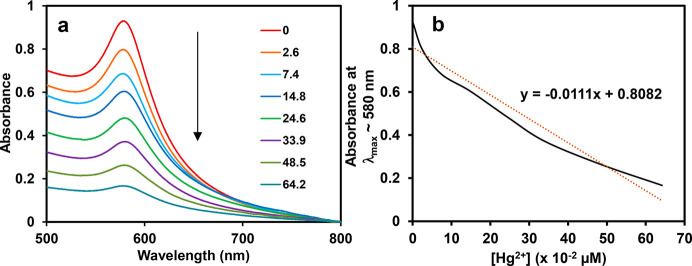
(a) UV–Vis spectra of DS-CuNP sol in the presence of varying concentration of Hg^2+^ (Legend: value × 10^–2^ μM). (b) Plot of maximum absorption of CuNPs at λ_max_ against [Hg^2+^].

The detection of metal ions using CuNPs involved a gradual attenuation of the SPR band of the NPs when the concentration of cations was increased. For instance, the absorption spectra obtained during additions of definite amounts of Hg^2+^ into a DS-CuNP solution (taken 3 mL) led to diminishing of the SPR band. The spectra so-obtained are displayed in Figure 5a. Upon progressively increasing the concentration of Hg^2+^ cation from 2.6 × 10^–2^ to 64.2 × 10^–2^ μM, the SPR band was found to undergo a steady fall marked by broadening of spectral lines.^29^ It may be mentioned that the concentration and hence the absorbance of the original DS-CuNP solution gradually kept on decreasing, upon increasing the Hg^2+^ concentration, on account of the rise in its volume from starting at 3 mL to ending at 8.4 mL. The disappearance of the SPR band was marked by a concomitant change from a reddish-brown color of the DS-CuNP colloid to milky-white. Further, predictably, a plot of the absorption at λ_max_ ≈ 580 nm of CuNP sol [values noted from Figure 5a], as a function of [Hg^2+^], exhibited a nearly linear relationship, as portrayed in Figure 5b. Initially, a steep decline in absorbance could be observed up to about 35 × 10^–2^ μM; however, after that point, the decline was slow. It could also be noted from the plot that the lowest Hg^2+^ concentration that was able to effect a change in CuNP absorbance was as small as 1 μM. Such a spectral change noticeable from UV–vis spectra, though small, is only of academic relevance, for no visual color change could be detected with a meager 1 μM Hg^2+^ concentration. The practical limit for the visual detection of color change of the original CuNP colloid was found to be that using 10 μM Hg^2+^. Additionally, on fitting a trendline to the data, a best-fit straight line with a negative value of slope was obtained. The declining slope and a gradual fall in absorbance is possibly indicative of the minuscule nature of changes brought about on every injection of Hg^2+^, in addition to the effect of dilution.

As an auxiliary experiment that was carried out to check if dilution alone were responsible for the drop in CuNP absorbance and the nature of the observed spectra, we added liquid water, sans Hg^2+^, to CuNP sol, and the spectra were recorded (Figure S2). The spectra so-obtained were found to be different from those in Figure 5a. Although a decline of the absorbance value of the CuNP solution (starting 3 mL) upon dilution is apparent, one could observe that the nature of the spectral lines on going from a spectrum corresponding to the concentrated solution to the one for a dilute solution remained the same. Besides, a rapid line broadening that was observed for a Hg^2+^-treated sample was missing in the dilution experiment spectra. Needless to mention that, in addition to the broadening of the band, the attenuation of the SPR peak was predominantly higher for the Hg^2+^-CuNP solution than for the dilute solution of CuNPs (results summarized in Table S1), when identical volumes of Hg^2+^ solution and water were added to CuNP colloid. Lastly, the color of the final dilute CuNP solution (8.4 mL) appeared bluish (possibly due to the oxidation of CuNPs to Cu^2+^), whereas, as mentioned above, Hg^2+^-CuNP solution eventually turned white. This suggests that Hg^2+^ indeed had a role in dampening the CuNP plasmon band and bringing about a change in the solution color.

It is also imperative to state here that a chemical reaction between mercuric ion and zerovalent copper atom—given that their differential reduction potential values permit so—is possible according to the following equation.
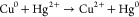


From the standard reduction potential values *E*^0^_Cu^2+^__/Cu_ = 0.34 V and *E*^0^_Hg^2+^__/Hg_ = 0.80 V (at 25 °C), taken from standard textbooks, the *E*^0^ was calculated to be *E*^0^ = 0.80 – 0.34 = 0.46 V. The positive value of *E*^0^ suggests that the above reaction is favorable. This certifies that the reduction in the absorbance value of CuNPs marked by a change in its color upon mixing with Hg^2+^ is due to the aforementioned redox reaction. Furthermore, formation of CuNP aggregates is also possible owing to the occurrence of chemical reactions in the medium. The formation of such aggregates could also be one of the reasons for the broadening of the spectral band.

**Figure 6:**
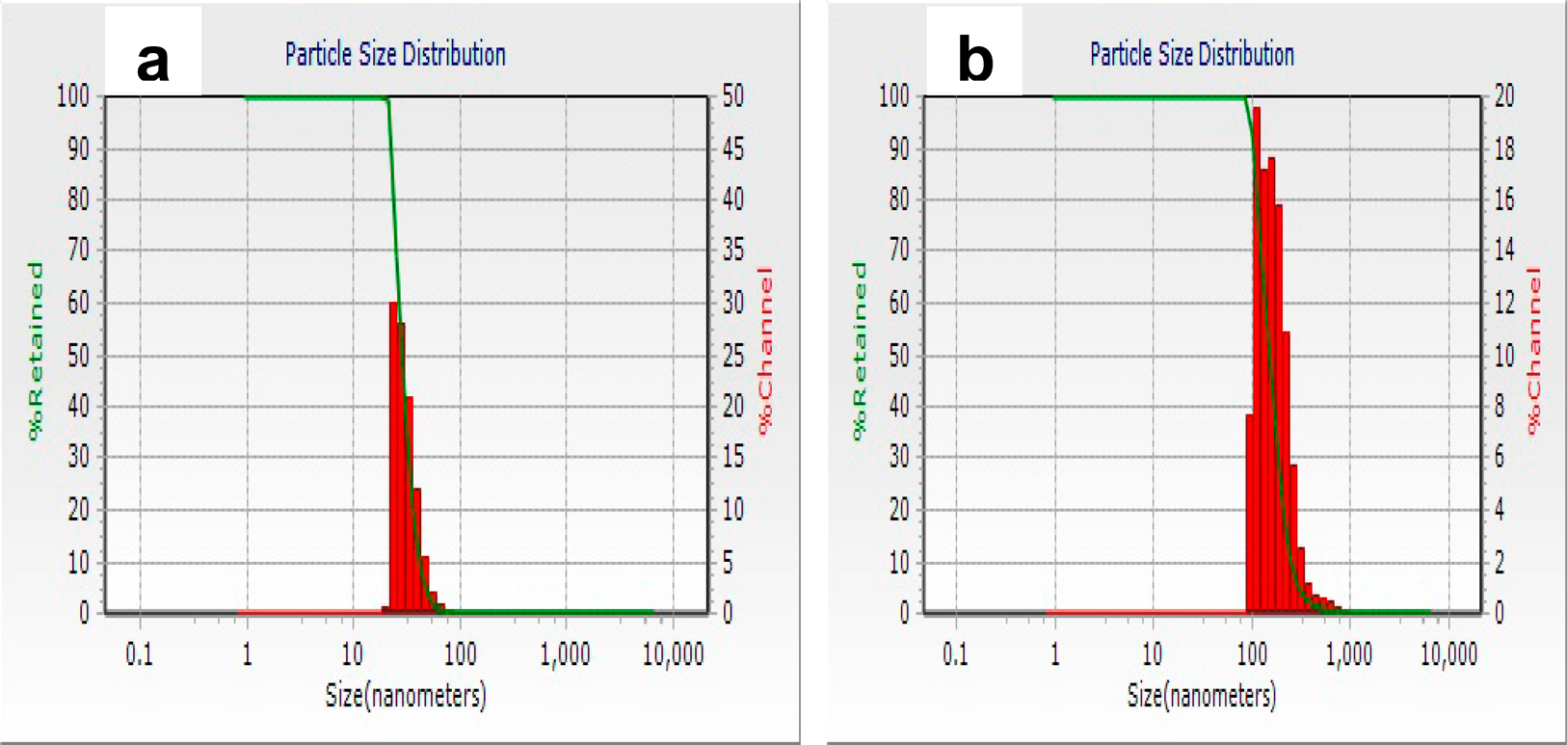
DLS profiles of DS-CuNPs (a) before and (b) after treatment with Hg^2+^.

Further, dynamic light-scattering (DLS) measurements were conducted to probe the size distribution of particles, particularly the hydrodynamic diameter of nanostructures. Figure 6a,b shows the particle size distribution plots of DS-CuNPs and Hg^2+^-treated DS-CuNPs. It may be mentioned here that the latter sample was the one prepared by having the highest [Hg^2+^] in the medium of CuNPs, as mentioned under UV–vis results. It could clearly be noticed that the DS-CuNPs possessed a hydrodynamic diameter of 20–30 nm. However, for the Hg^2+^-DS-CuNP sample, the hydrodynamic diameter was observed at 200–250 nm. This hints at the possible formation of aggregates of CuNPs upon mixing DS-CuNPs with Hg^2+^. DLS spectra of control samples consisting of SDS-Hg^2+^ solution and pure SDS solution, provided in Figure S3a,b, showed the size distribution for both samples as 1 nm. These supporting data helped us to figure out that no aggregates had formed between a mixture of SDS micelles and Hg^2+^ and that the size of SDS-Hg^2+^ is the same as pure SDS. Hence, it could be stated that the observations from DLS studies were in accord with UV–vis studies so long as the formation of aggregates of CuNPs in the presence of mercury cation is considered.

**Figure 7:**
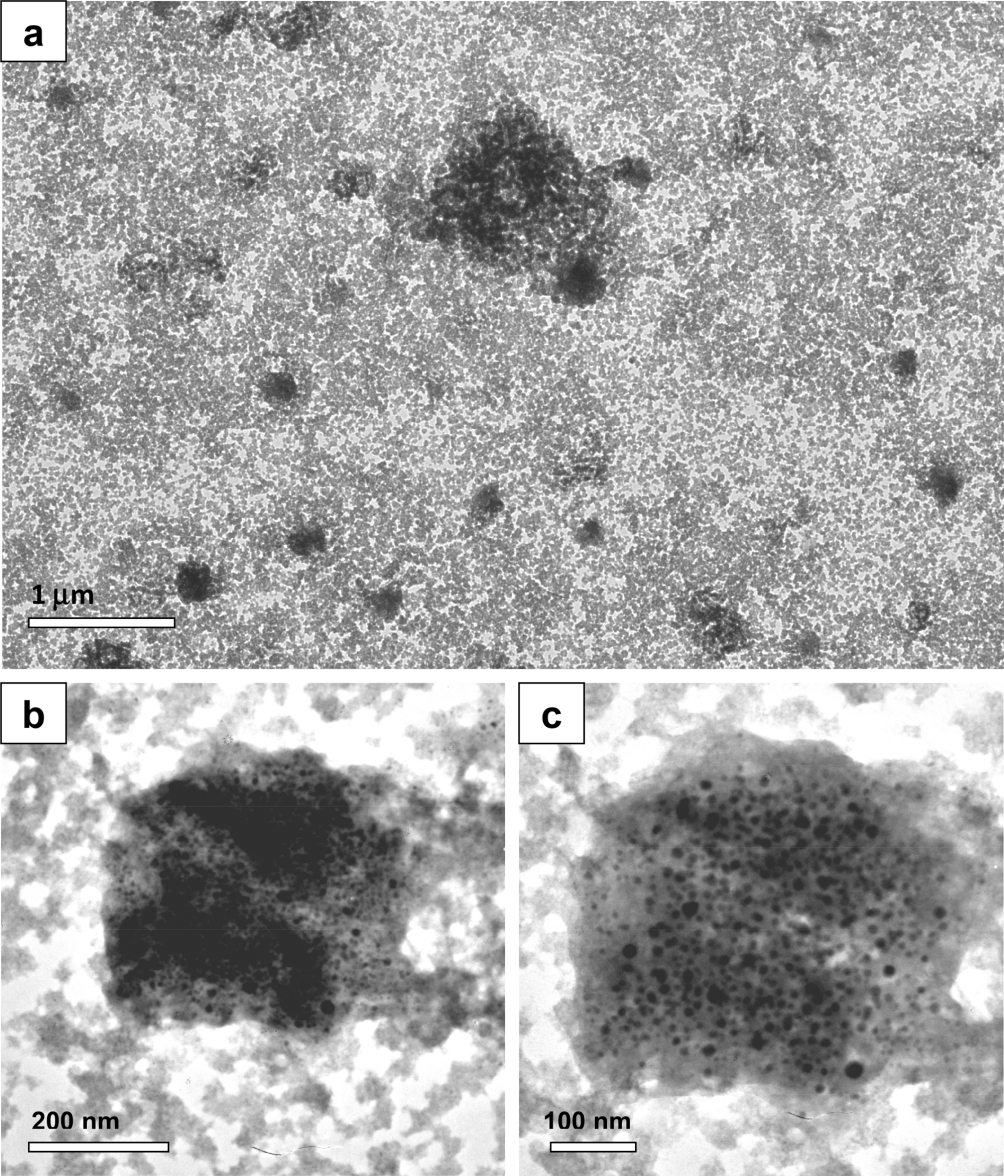
(a–c) TEM images of a dispersion of Hg^2+^-treated DS-CuNPs. (a) Large area view, (b) a portion of NP assembly, and (c) an enlarged view of (b).

The results of DLS measurements were corroborated by a TEM investigation of an Hg^2+^-treated DS-CuNP solution (with highest [Hg^2+^], mentioned above). It could be observed from Figure 7a that a large number of structures, mostly spherical in shape and sizes on the order of 250 nm or higher, had formed in the solution. These superstructures, on magnification, revealed the existence of tens of hundreds of NPs in close proximity to each other [Figure 7b,c], akin to NP agglomerates, but with the exception that the NPs are somewhat detached from each other, giving unaggregated structures. It seems also that some particles underwent coalescence with others, forming somewhat bigger particles here and there. We conjecture that it could be because of the presence of these unaggregated superstructures and larger particles in the medium that broadening of the UV–vis band might have occurred, progressively with the rise in [Hg^2+^] in CuNP solution. This also justifies the cause for the nonevolution of a band or shoulders in the longer wavelength region, an observation identical to that of a previously reported study.^29^ Thus, UV–vis, DLS, and TEM measurements collectively confirm the formation of superstructures or aggregates consisting of CuNPs as the building blocks, when in the presence of Hg^2+^.

**Figure 8:**
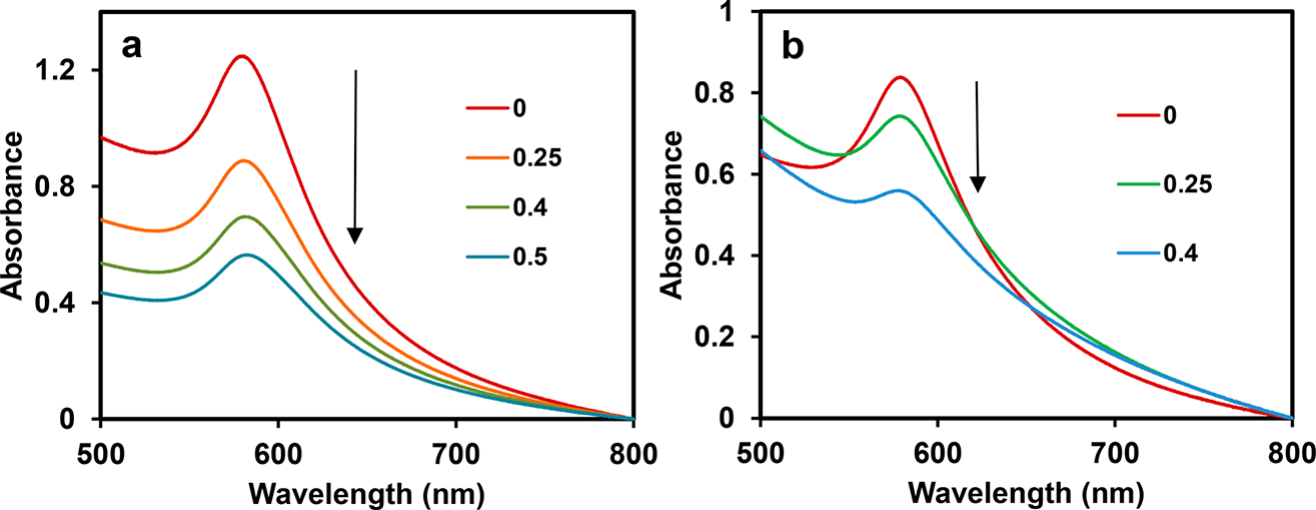
UV–Vis spectra of DS-CuNPs in the presence of (a) Cd^2+^ and (b) Pb^2+^ solutions of different concentrations (Legend: concentrations of metal ions expressed in μM units).

Identical to the detection of Hg^2+^, the experiments relating the determination of Cd^2+^ and Pb^2+^ ions revealed the diminishing of the SPR band of DS-CuNPs when the concentration of the metal ions was increased. The results of the analyses involving the two cations are presented in Figure 8. It is noticed from the figure that the amounts of metal ions required for affecting the SPR is way higher than that using Hg^2+^. Further, physical interactions between the cationic species (Cd^2+^, Pb^2+^) and CuNPs might be a possible reason for the resultant change in the NP SPR band, unlike Hg^2+^, which is able to chemically react with CuNPs.

**Figure 9:**
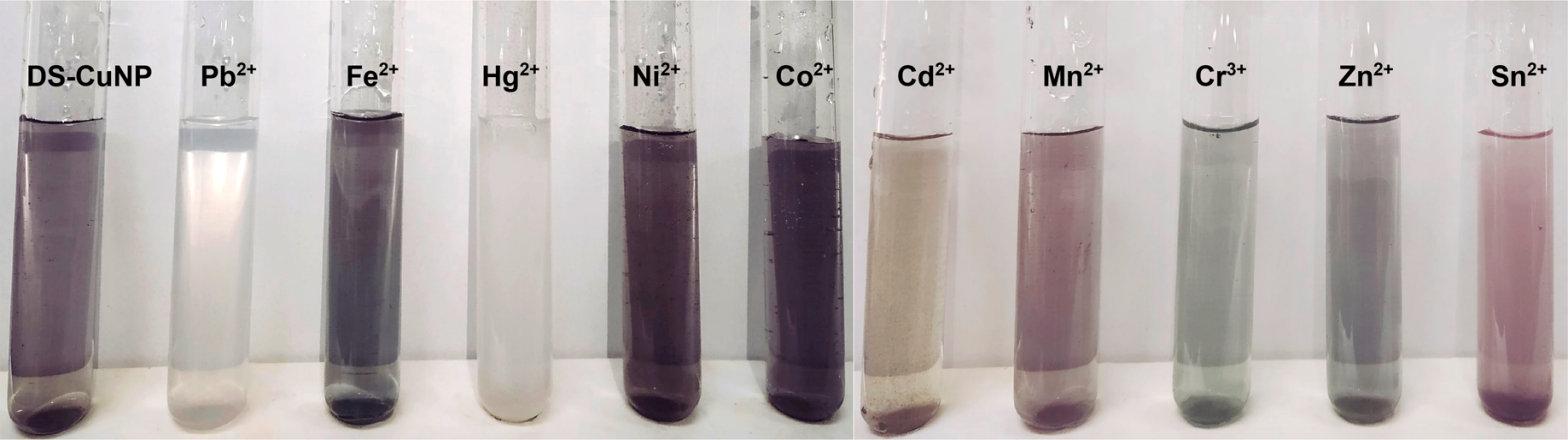
Photograph of DS-CuNP solution after being treated with different metal ions (conc ≈ 10 μM).

In both the cases involving Pb^2+^ and Cd^2+^ ions, precipitation of CuNPs resulted in a final observation of solution color—white or greyish-white, as shown in Figure 9. The same figure also depicts the photographs of solutions corresponding to treatment of DS-CuNPs with a series of other heavy metal ions. All in all, the colorimetric detections of the ions Hg^2+^, Pb^2+^, and Cd^2+^ were prominent using CuNPs. The limit of detection was found to be 10 μM for Hg^2+^ and relatively higher for Cd^2+^/Pb^2+^.

**Figure 10:**
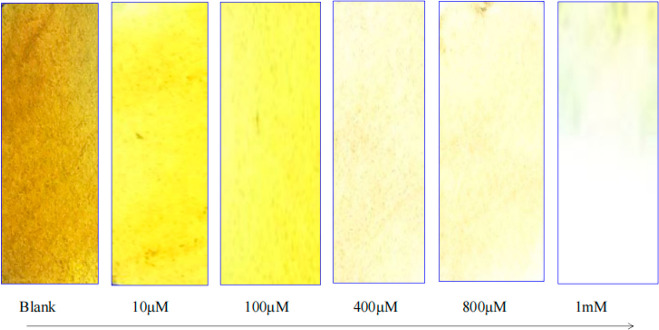
Test paper strips treated with a range of concentration of Hg^2+^ solution.

Interestingly, extending the sensing experiment of Hg^2+^ using DS-CuNPs suspended on a paper led to remarkable results. Precisely, test paper strips containing CuNPs, when dipped into different heavy metal ion solutions (Hg^2+^, Cd^2+^, Pb^2+^, Zn^2+^, Ca^2+^), showed no significant changes for the metal solutions of Cd^2+^, Pb^2+^, Zn^2+^, and Ca^2+^. However, when a few drops of Hg^2+^ were added to the brownish-yellow test paper strip, decoloration of the paper resulted in a spectrum of colors ranging from yellow to pale yellow and milky-white, by varying [Hg^2+^] (Figure 10 & Figure S4). As mentioned above, a redox reaction between Hg^2+^ and Cu^0^ is possibly responsible for bringing about a color change to the paper strip, on exposure to a Hg^2+^-containing solution. In stark contrast, the reduction potential values of Cd^2+^, Pb^2+^, Zn^2+^, and Ca^2+^ are lower than those of Cu^2+^/Cu, for which auto oxidation–reduction is not favorable. It is for this reason that these ions fail to affect any color change to the paper strips. However, it might be possible that, at higher concentrations, these ions could change the color of paper strips. But, that change would be ascribed to physical interactions between the metal ions and CuNPs and not because of a chemical reaction.

## Conclusions

We have reported the synthesis of a stable CuNP dispersion by a chemical reduction method using dodecyl sulfate ion as a surface passivating agent. The particles were characterized using routine techniques like UV–vis spectroscopy, Fourier transform infrared spectroscopy, dynamic light scattering, and transmission electron microscopy. The as-prepared DS-CuNP colloid was treated with a host of heavy metals, which indicated that DS-CuNPs are highly selective and sensitive toward Hg^2+^ ions. The detection limit was in the range of 10 μM. Further, test paper strips containing the CuNPs facilitated the detection of Hg^2+^ by a visual change in its color. The method is robust in that it involves the use of a low-cost, easy detection and easily disposable paper strips. We believe that this method would offer a new approach for the detection of heavy metal ions in aqueous, biological, and environmental samples, for an evolving point-of-care detection of toxic metal ions.

## Supporting Information Available

Characterization methods, TEM images, UV–vis spectra, DLS profiles of samples, and experimentation scheme for Hg^2+^ sensing (PDF)
