# Supplementary material for: Cu Nanoparticle-Based Solution and Paper Strips for Colorimetric and Visual Detection of Heavy Metal Ions
Source: ACS Omega. 2022 Oct 9;7(42):37279–85. doi: 10.1021/acsomega.2c03687 (PMC9609079; doi:10.1021/acsomega.2c03687)
Supplement: Supplementary file 1 — ao2c03687_si_001.pdf [file ao2c03687_si_001.pdf]

# Supporting Information

# Cu Nanoparticle-Based Solution and Paper Strips for Colorimetric and Visual Detection of Heavy Metal Ions

Trilochan Baral,[1] Chitraniva Datta,[1] and Subhojit Das[1*]

[1]Department of Chemistry, National Institute of Technology Agartala, Tripura-799046, India.

Email: sdas.chem@nita.ac.in

## Characterization Methods

The optical properties like band gap, absorption maxima etc., of the DS-CuNPs were monitored on Shimadzu UV-2600i UV-Vis Spectrophotometer. Fourier transform infrared (FT-IR) spectra were recorded in the wavenumber range of 4000 to 500 $cm^{-1}$ - in order to investigate the mechanism of conjugation of ligand with NPs - using Perkin Elmer Spectrum Two Spectrometer by initially making a disc of the sample with KBr. The hydrodynamic diameter and size dispersion of NPs in the aqueous media were determined using a Microtrac *Nanotrac Wave* particle size analyzer. Field Emission Transmission Electron Microscope (JEOL, JEM-2100F operating at an accelerating voltage of 200 kV) was used to determine the morphology and crystallinity of the NPs. TEM samples were prepared by air-drying a drop of a solution containing NPs on a 300 mesh carbon-coated Cu grid.

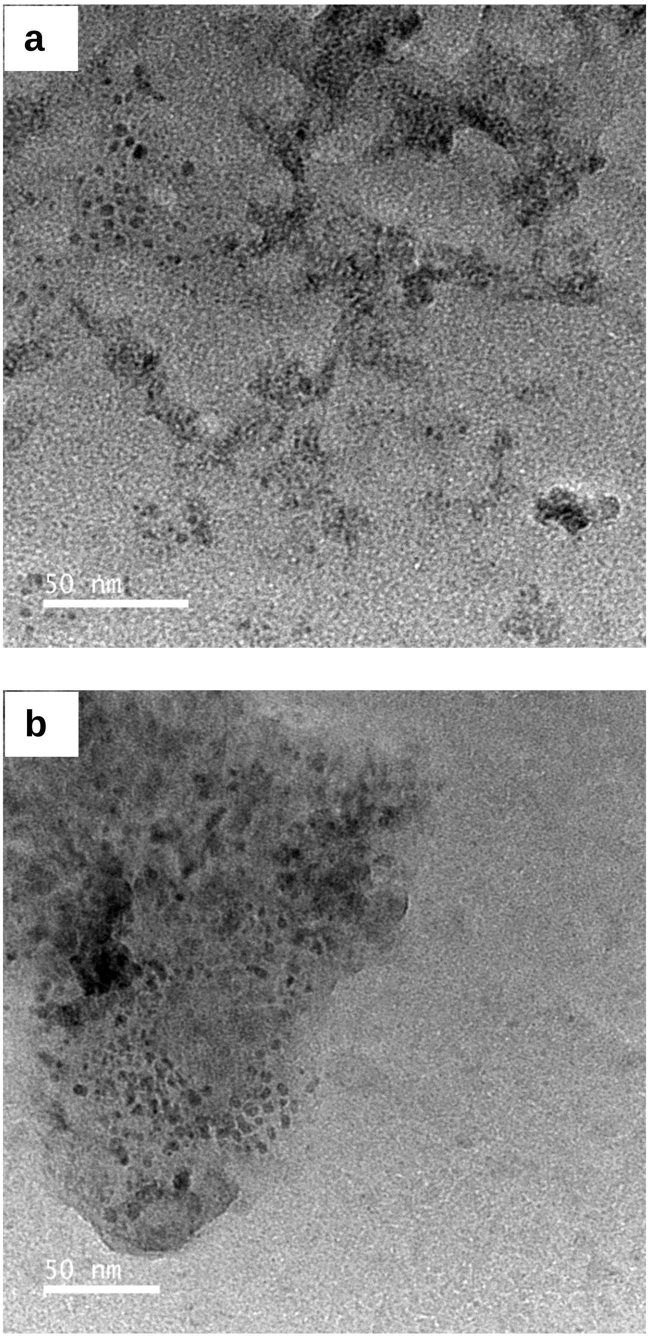


**Figure S1.** (a-b) TEM images of as-synthesized DS-CuNP dispersion.

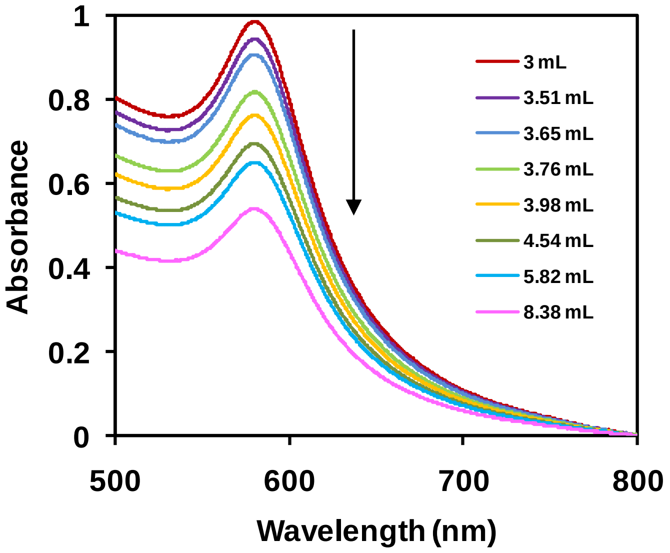


**Figure S2.** UV-Vis spectra of DS-CuNP dispersion on serial dilution with liquid water. (Legend: volume of water used for the dilution of initially 3 mL CuNP solution.)

## Table S1

### Effect of $Hg^{2+}$ addition/aqueous dilution on the SPR of CuNPs: Comparative Analyses

| DS-CuNP sol (A) | Addition of 1 μM $Hg^{2+}$ solution to 'A' (I) | | Water volume used for dilution of 'A' (mL) (II) | Observed absorbance value (SPR) due to | | Net attenuation of SPR w.r.t. initial spectrum of 'A' | |
|---|---|---|---|---|---|---|---|
| | **Vol. added (mL)** | **Net conc. (× $10^{-2}$ μM)** | | **I** | **II** | **I** | **II** |
| 3 mL (fixed) | 0 | 0 | 0 | 0.93 | 0.98 | 0 | 0 |
| | 0.08 | 2.6 | 0.51 | 0.79 | 0.94 | 0.14 | 0.04 |
| | 0.24 | 7.4 | 0.65 | 0.68 | 0.90 | 0.25 | 0.08 |
| | 0.52 | 14.8 | 0.76 | 0.60 | 0.81 | 0.33 | 0.17 |
| | 0.98 | 24.6 | 0.98 | 0.48 | 0.76 | 0.45 | 0.22 |
| | 1.54 | 33.9 | 1.54 | 0.37 | 0.69 | 0.56 | 0.29 |
| | 2.82 | 48.5 | 2.82 | 0.26 | 0.65 | 0.67 | 0.33 |
| | 5.38 | 64.2 | 5.38 | 0.17 | 0.54 | 0.76 | 0.44 |

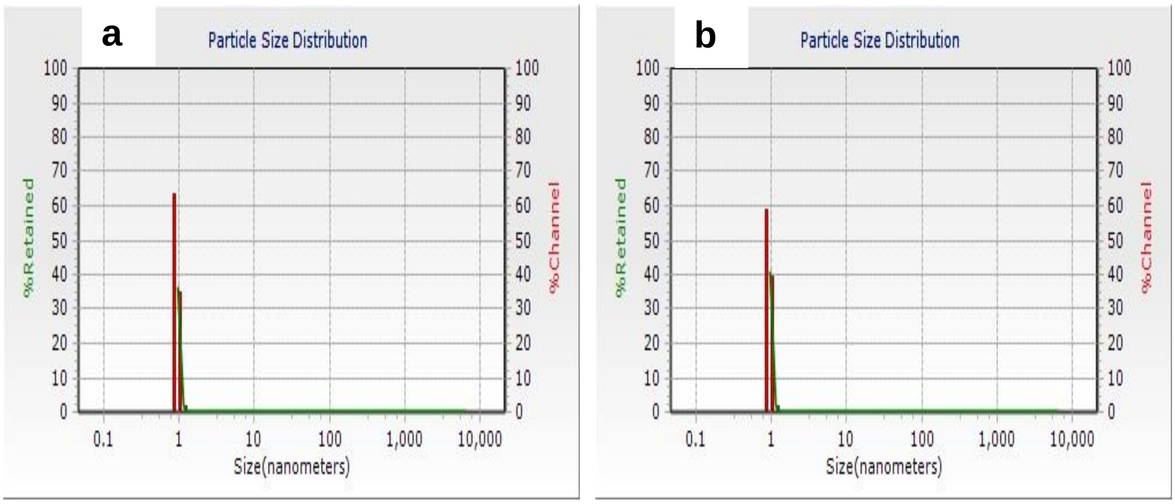


**Figure S3.** DLS profiles of solutions of (a) SDS-$Hg^{2+}$ and (b) SDS.

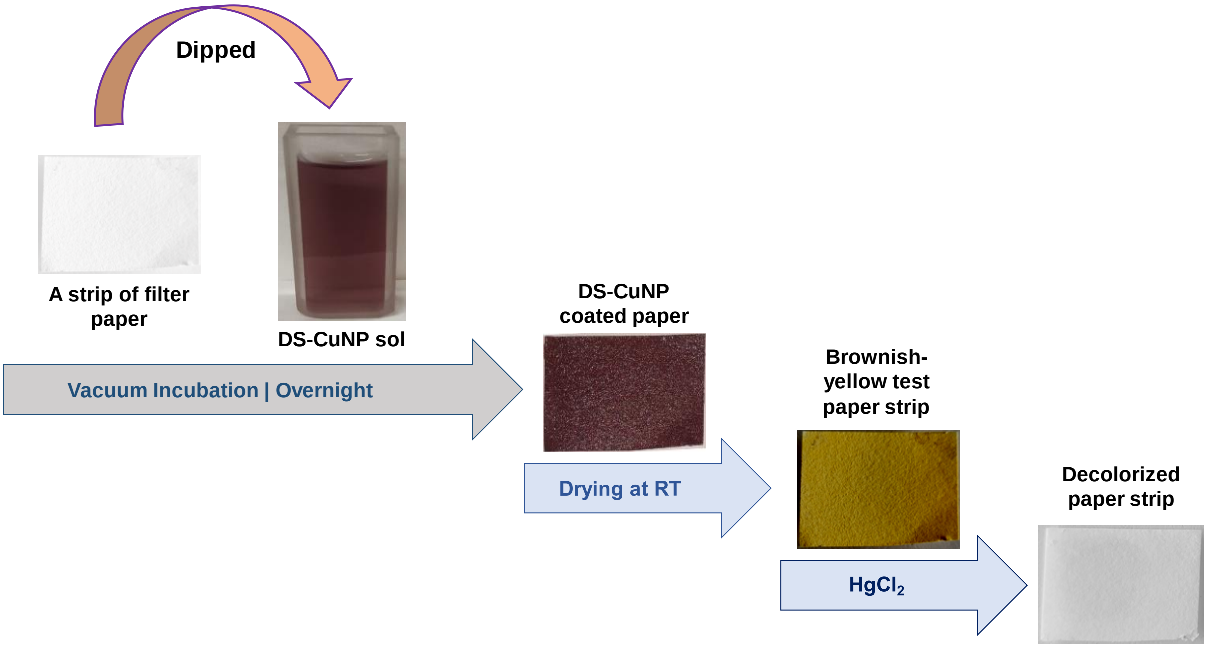


**Figure S4.** Pictorial representation of the steps involving the coating of DS-CuNPs on a strip of filter paper and its subsequent use for the detection of $Hg^{2+}$.
